# Embelin Prevents Seizure and Associated Cognitive Impairments in a Pentylenetetrazole-Induced Kindling Zebrafish Model

**DOI:** 10.3389/fphar.2019.00315

**Published:** 2019-04-17

**Authors:** Uday Praful Kundap, Yam Nath Paudel, Yatinesh Kumari, Iekshan Othman, Mohd. Farooq Shaikh

**Affiliations:** ^1^ Neuropharmacology Research Laboratory, Jeffrey Cheah School of Medicine and Health Sciences, Monash University Malaysia, Bandar Sunway, Malaysia; ^2^ University of Montreal Hospital Centre (CRCHUM), Montreal, QC, Canada

**Keywords:** chronic epilepsy, zebrafish, three-axis maze, cognition, behavior

## Abstract

Epilepsy is a neuronal disorder associated with several neurological and behavioral alterations characterized by recurrent spontaneous epileptic seizures. Despite having more than 20 anti-epileptic drugs (AEDs), they only provide a symptomatic treatment. As well as, currently available AEDs also displayed cognitive alterations in addition to retarding seizure. This leads to the need for exploring new molecules that not only retard seizure but also improve cognitive impairment. Embelin (EMB) is a benzoquinone derivative which has already demonstrated its pharmacological potentials against arrays of neurological conditions. The current study developed a chronic kindling model in adult zebrafish by using repeated administration of small doses of pentylenetetrazole (PTZ) and a single dose of Kainic acid (KA) to investigate the associated memory impairment. This has been done by using the three-axis maze which is a conventional method to test the learning ability and egocentric memory in zebrafish. As well as, the ameliorative potential of EMB has been evaluated against chronic epilepsy-related memory alterations. Moreover the expression level of pro-inflammatory genes such as C-C motif ligand 2 (CCL2), toll-like receptor-4 (TLR4), tumor necrosis factor-α (TNF-α), interleukin-1 (IL-1) and interferon-γ (IFN-γ) were evaluated. The level of several neurotransmitters such as γ-aminobutyric acid (GABA), acetylcholine (Ach) and glutamate (Glu) was evaluated by liquid chromatography-mass spectrometry (LC-MS). The results showed that daily dose of PTZ 80 mg/kg for 10 days successfully induces a kindling effect in zebrafish, whereas the single dose of KA did not. As compared to control, the PTZ and KA group demonstrates impairment in memory as demonstrated by the three-axis maze. The PTZ group treated with a series of EMB doses (ranging from 0.156 to 0.625 mg/kg) was found to have retarded seizure as well as significantly reduces epilepsy-induced memory alteration. In addition, EMB treatment reduces the expression of inflammatory markers implicating its anti-inflammatory potential. Moreover, levels of GABA, Ach, and glutamate are increased in EMB administered group as compared to the PTZ administered group. Overall, findings demonstrate that EMB might be a potential candidate against chronic epilepsy-related cognitive dysfunction as EMB prevents the seizures, so we expect it to prevent the associated neuroinflammation and learning deficit.

## Highlights

- A repeated small dose of PTZ (80 mg/kg) (kindling) for 10 days produces chronic epilepsy like condition in adult zebrafish.
- EMB was reported to reduce epileptic seizures and improve long term memory as evidenced by a three-axis maze test.
- EMB demonstrated an anti-inflammatory effect *via* downregulating several inflammatory markers (CCL2, TLR4, IL-1, and IFN-γ) in the epileptic brain.
- EMB might be a potential candidate against chronic epilepsy and related cognitive dysfunction as well as can overcome the limitations of mainstream AEDs.

## Introduction

Epilepsy is a neurologic disorder affecting more than 70 million people worldwide (Singh and Trevick, 2016). Epilepsy is mainly characterized by the occurrence of spontaneous recurrent seizures (SRS) and high prevalence of comorbidities, such as cognitive impairments, depression, and anxiety, affecting the lives of individuals (Keezer et al., 2016). Chronic epilepsy is characterized by repeated unpredictable seizures, impairing memory in 30–40% of patients under long-term anti-epileptic drugs (AEDs) treatment, so epilepsy and AEDs both are responsible for memory problems (Helmstaedter, 2002; Canevini et al., 2010; Zhang et al., 2017). Despite there being more than 20 mainstream AEDs, they only provide symptomatic treatment rather than interfering with the disease’s mechanism, and 30% of patients do not respond to current AEDs (Remy and Beck, 2005; Chen et al., 2018). These data indicate the pressing need to develop novel and innovative therapy that can reduce seizures and maintain good memory status. Despite recent advances in research, the underlying pathomechanism of epilepsy is still less known. However, findings are emerging that implicate the role of inflammation in epilepsy (Vezzani, 2005; van Vliet et al., 2018; Paudel et al., 2018b). High mobility group box 1 (HMGB1) protein is an initiator and amplifier of neuroinflammation and has been implicated in seizures *via* activating toll-like receptor 4 (TLR4) (Ravizza et al., 2017; Paudel et al., 2018a). Moreover, C-C motif ligand 2 (CCL2), tumor necrosis factor-α (TNF-α), interleukin-1 (IL-1) and interferon-γ (IFN-γ) have been implicated in seizure generation as well as found to be up-regulated during a seizure (Marchi et al., 2014; Dey et al., 2016).

Local or systemic administration of kainate in rodents leads to repetitive limbic seizures and status epilepticus (SE), lasting for several hours. It is a useful model of temporal lobe epilepsy (TLE) in rodents (Dal-Pizzol et al., 2000). Kainic acid (KA) is an epileptogenic and the neuroexcitotoxic agent acting on specific kainate receptors (KARs) in the central nervous system (CNS) (Zheng et al., 2011). The complete seizure characterization of KA-induced epilepsy is studied in zebrafish and it produces continuous seizures with neuronal toxicity (Alfaro et al., 2011; Menezes et al., 2014; Mussulini et al., 2018). Traditional models like the maximal electroshock seizure (MES) and pentylenetetrazole (PTZ) seizure models have been instrumental for pre-clinical screening for a long time, however, these models have limited themselves in identifying novel compounds with improved efficacy against different chronic disorders (Löscher, 2011). A PTZ induced acute seizure model has been already developed in adult zebrafish and recapitulates all the clinical phenotypes of seizure (Mussulini et al., 2013; Kundap et al., 2017). Kindling is a chronic animal model of epilepsy that has been extensively studied to understand the underlying mechanism of epileptogenesis (Kandratavicius et al., 2014). In addition, kindling is a phenomenon where a sub-convulsive stimulus (either chemical or electrical), if applied repetitively and intermittently, will ultimately lead to the generation of full-blown convulsions (Dhir, 2012a). The study was conducted by using PTZ kindling discusses detail methodology to execute a chemical induced kindling in mice (Dhir, 2012a). A number of different promising models which fulfill this criterion have been developed over recent years, but has not been modeled in zebrafish (Wilcox et al., 2013). Moreover, the understanding of the molecular involvement underlying such diseases is still under study and it is preventing adequate therapeutic outcomes (Samarut et al., 2016). Various numbers of pre-clinical models have been studied to investigate the role of different brain functions and understand the structure of disease development (Samarut, 2016).

In recent days, zebrafish disease models has received increased attention due to its genetic similarity to humans, economic value, and its suitability as an alternative for a human disease model and for large-scale drug screenings (Shams et al., 2018). Zebrafish has rapid embryonic development as compared to rodents, as well as easy cellular observation and phenotypic analysis, which makes it a better model to study the neurological aspect of a brain disorder.

The three-axis maze is a method to evaluate learning ability and egocentric memory in zebrafish. In a three-axis maze, fish are required to navigate a route based on x (forward/backward), y (horizontal) and z (vertical) axes (Nasir et al., 2012). Egocentric navigation is an evidence-based process, where fish navigate to the feeding ring independent of environmental cues and deals directly with the spatial relationship between subject and reward goal (Nasir et al., 2012).

Embelin (EMB) (2,5-dihydroxy-3-undecyl-1,4 benzoquinone) is the main component in the red berry fruits produced by *Embelia ribes* (Dubéarnès et al., 2015). EMB has been studied extensively using various in-vitro and in-vivo animal models and have exhibited strong anti-convulsant, anxiolytic and anti-depressant properties as well as improving conditions such as sickness behavior, Huntington’s disease (HD), multiple sclerosis (MS), cerebral ischemia and traumatic brain injury (TBI) (Mahendran et al., 2011; Poojari, 2014). Moreover, earlier findings reported that EMB significantly inhibited seizures induced by electroshock and PTZ in a dose-dependent manner (Shankar et al., 2012). In spite of the huge pharmacological significance of EMB, no study has earlier reported the ameliorative potential of EMB against PTZ kindling induced chronic epilepsy and related cognitive alterations using zebrafish as an animal model.

The current investigation developed a protocol of PTZ kindling in an adult zebrafish. A small amount of proconvulsant drug (PTZ) was administered for a period of 10 days in order to produce progressive chronic epilepsy like behavior. To KA-treated fish, as per rodent model, we tried to induce chronic epilepsy by administrating a single dose of KA-3 mg/kg the day before the start of the experiment. The seizure scores were calculated, and the three-axis maze trial was performed in order to check the memory status and seizure progression on a daily basis. The same procedure was employed in the fish pretreated with EMB and the effect of EMB was analyzed. In addition, fold change of neuroinflammatory genes and neurotransmitter levels were quantified in order to confirm the positive effect of EMB against chronic epilepsy induced cognitive dysfunction model in adult zebrafish.

## Materials and Methods

### Experimental Equipment and Chemicals List

All analytical grade reagents were used unless specified otherwise. Water was purified and filtered with a specific liquid chromatography-mass spectrometry (LC-MS) filter using a Milli-Q system from Millipore (Bedford, MA, USA). Glutamate, γ-aminobutyric acid (GABA), KA, and PTZ were purchased from Sigma Aldrich (USA). The pure form of plant extract of EMB was purchased from YUCCA Enterprises, Mumbai, India. Ethanol 95% (EtOH) was purchased from Kolin Chemicals Co. Ltd, Korea, methanol (MeOH), chloroform (CHCl3), isopropanol (IPA), and formic acid (FA) was purchased from Friedemann Schmidt Chemicals, Parkwood 6147, Western Australia.

### Zebrafish Maintenance and Housing Conditions

Adult zebrafish (*Danio rerio*) of heterozygous wild-type-AB stock (standard short-fin phenotype) were obtained from the Institute of Molecular and Cell Biology (IMCB), 61 Bioplis Drive Proteos, Singapore 138673. All fish were kept in the Monash University Malaysia fish facility at 28°C, with a 10/14 h dark/light cycle (white incident light off at 10 pm, white incident light on at 8 am) under standard aquarium conditions. Care was taken to maintain system water pH between 6.8–7.1 by using an electronic pH pen (Classic PH Pen Tester, Yi Hu Fish Farm Trading, Singapore 698950) and intensity of light was maintained at 250 Lux to get the uniform light all over the housing area. Fish were fed, thrice a day to ensure a constant source of nourishment. Nutrition for fish was maintained by Tropical TetraMin® Flakes and live brine shrimps Artemia from Bio-Marine (Aquafauna, Inc. United States). Circulating water system with zebrafish tank which is equipped with constant aeration having (36 × 26 × 22 cm) tank dimensions (Kundap et al., 2017). Monash Animal Research Platform (MARP), Australia, approved all the zebrafish experimental procedures (MUM-2017-03) (MARP-2017-003).

During the experimental procedure the animals were housed individually in each tank of 3 L in size. The animals were not fed in the home tank as they received the food a reward during each trial daily. The fish that did not reach the feeding ring (goal chamber) were fed once in the home tank. To avoid the encounter of aggressive behavior with each other and to individually track the behavior of each fish individually, the fish were housed separately in each tank (Parker et al., 2012). The water temperature of 28°C was maintained the same as the system water, with 7.2 pH, the light–dark cycle of 14/10 h, and intensity of light was maintained at 250 Lux to get uniform lighting all over the housing area.

### Animal Grouping/Randomization and Treatment

**Figure 1:**
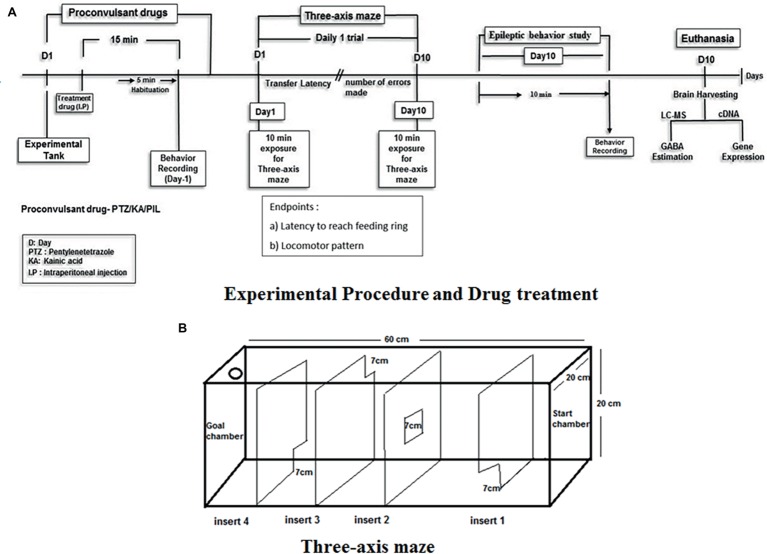
Experimental setup and design procedure. **(A)** Represents the flowchart represents the scheme for EMB treatment, PTZ administration and behavior recording for epilepsy and three-axis maze used in the study. **(B)** Acrylic white experimental box of Three-axis maze with different openings from start chamber to goal chamber (feeding ring).

Groups of animals exposed to drug treatment were divided as follows. Animals selected for testing were randomized as per the groups with *n* = 12. The experiment was not blinded as videos were recorded and then manually analyzed and checked by two investigators individually for confirmation. The fish were pre-treated with embelin and then exposed to PTZ before the start of the three-axis maze trial. The behavior recording for an epilepsy seizure score and transfer latency was recorded daily for the period of 10 days for each fish. Animal treated with embelin were observed for its epilepsy behavior, cognitive performance in three-axis maze and biochemical changes. Later on, the zebrafish brains were isolated for LC-MS/MS studies and gene expression analysis. For experimental procedure and drug administration follow Figure 1.

Set 1: Development of chronic epilepsy induced cognitive problems in zebrafish.

**Group I:** Vehicle control; **Group II:** PTZ 80 mg/kg; **Group III:** KA 3 mg/kg.

**Set 2:** Elucidating the therapeutic potential of EMB against chronic epilepsy induced cognitive dysfunction.

EMB and PTZ were dissolved in 10% DMSO.

**GroupI:** Vehicle control (10% DMSO); **GroupII:** Pentylenetetrazole (PTZ)–80 mg/kg; **Group III:** Embelin (EMB)–0.156 mg/kg + PTZ; **Group IV:** Embelin (EMB)–0.312 mg/kg + PTZ; Group V: **Embelin (EMB)**–0.625 mg/kg + PTZ.

### Software and Equipment

The Smart V3.0.05 tracking software (Pan Lab, Harvard Apparatus), the Sony (Handycam HDR-PJ340E) video camera and Sony Camcorder stands were used for the automated tracking of zebrafish swimming patterns and locomotion parameters. The Applied Biosystems StepOnePlusTM Real-Time PCR System was used for the gene expression study. The MilliQ system from Millipore (Bedford, MA, United States) was used to produce pure water for the experiment purpose. The equipment used for quantifying brain neurotransmitters was the Agilent 1,290 Infinity UHPLC, coupled with the Agilent 6410 Triple, Quad LC/MS. Fish tank −10 liters capacity (PETCO-Pet keeper, Malaysia), The syringe used for intraperitoneal injection was Hamilton syringe 700–702 series 25 μl along with BD disposable needle–30G (Becton Dickinson, USA).

### Procedure for Zebrafish Anesthesia and Intraperitoneal Injection

PTZ and the EMB were intraperitoneally infused into the zebrafish as indicated by the convention given by Kundap et al. (2017) and is given beneath. At the point when different intraperitoneal infusions were required, the infusions were given at alternating lateral ends, instead of the midline between the pelvic fins. Each zebrafish was caught individually utilizing a fish holding net, and after that moved into an anesthesia arrangement (30 mg/L Benzocaine). The fish were kept into the anesthesia water for 30 s until they stop moving. The zebrafish was taken out once anesthetized and weighed afterward to calculate the dose and subsequently the infusion volume. A delicate sponge roughly 20 mm in stature was soaked with water and set inside a 60 mm Petri dish. A cut between 10 and 15 mm inside and out was made in the sponge to control and hold the fish for the intraperitoneal infusion. The intraperitoneal infusion was given while utilizing a dismembering magnifying lens by embeddings the needle into the midline between the pelvic fins. An appropriate volume was then injected into the zebrafish, after considering the body weight of the zebrafish.

All intraperitoneal infusions were administered into the stomach pit at an area back to the pelvic support, utilizing a 10 μl Hamilton syringe (700 arrangement, Hamilton 80400) (Stewart et al., 2011). The experiment was performed in a separate behavior room with the room temperature kept between 26 and 30°C and humidity between 50 and 60%. All zebrafish were acclimatized in the said behavior room for 2 h prior to the experiment for minimizing any novel tank response. Other precautions taken include using a small injection volume of 10 μl per gram of fish and using a 30-gauge needle. The zebrafish were restrained in water saturated sponge under benzocaine anesthesia to reduce the distress inflicted on the zebrafish (Barbosa et al., 2012). This intraperitoneal injection technique was found to be effective in zebrafish (Kundap et al., 2017) and did not cause any mortality throughout the experiment. After the intraperitoneal injection, the zebrafish was immediately transferred to an observation tank.

### Seizure Score Analysis

#### PTZ-Induced Seizure Score

PTZ is a proconvulsant drug. As per earlier reported studies, PTZ at 220–250 mg/kg dose produces full-blown seizures in zebrafish (Banote et al., 2013). In order to produce kindling effect, PTZ at lower doses (80 mg/kg) was administered daily for 10 days which was almost 1/3 of the actual dose reported earlier. Kindling is a process by which a seizure or other brain event is initiated and its recurrence is made more likely (Dhir, 2012). The PTZ Kindling model is well established in rodents and in other animals so in accordance to those animal model the dose of PTZ administration is calculated for zebrafish (80 mg/kg dose/10 days) (Ohno et al., 2010). After the fish was prone to seizures for at least 10 days it was observed for its locomotor behavior, three-axis maze memory analysis, and other biochemical alterations. For the EMB treated group fish were pre-treated with a single dose of EMB dissolved in 10% DMSO prior to daily PTZ administration. However, in the control group fish were pre-treated with 10% DMSO and the distilled water was administered to maintain the same number of injection in all the groups.

PTZ injected zebrafish displays unique seizure characters, intensities and latency in generating the different seizure scores. PTZ induced seizure behavior will remain around 10 min after the PTZ administration and gradually decrease with time. The PTZ injected adult zebrafish were then transferred to the observation tank water for behavior and the three-axis maze test. The behavior of the zebrafish was then recorded for 10 min after recovery from anesthesia and the video was later viewed using a computer to determine the highest seizure score during the 10 min. The zebrafish seizure score was recorded as per the earlier scoring protocol (Banote et al., 2013; Mussulini et al., 2013; Kundap et al., 2017) and is given below.

Score 1–Short swim mainly at the bottom of the tank.

Score 2–Increased swimming activity and high frequency of opercula movement.

Score 3–Burst swimming, left and right movements as well as the erratic movements.

Score 4–Circular movements.

Score 5–Clonic–tonic full body system rhythmic contractions.

Score 6–Fall at the bottom of the tank.

Score 7–Death

#### KA-Induced Seizure Score

Throughout the time of pre-exposure to KA, the behavior of the zebrafish was monitored for 10 min after the fish was fully recovered from anesthesia. The seizure behavior for KA-induced seizure was modified from the earlier findings (Menezes et al., 2014) and described below.

Score 1–Rigidity and hyperventilation of the animal.

Score 2–Whirlpool-like swimming behavior.

Score 3–Rapid muscular uncontrol movements from right to left.

Score 4–Abnormal and spasmodic muscular contractions.

Score 5–Rapid whole-body clonus-like convulsions.

Score 6–Sinking to the bottom of the tank and spasms for several minutes.

Score 7–Death.

Under the directives of the Monash Animal Research Platform (MARP)-Australia, the PTZ dose was standardized at 80 mg/kg of zebrafish body weight in order to produce the kindling seizure progression. The dose of KA-3 mg/kg (single dose) (Alfaro et al., 2011) was titrated in order to produce the maximum survival of zebrafish in a group for more than 48 h until day-10. The highest observed seizure score was the highest seizure score noted within the entire 10 min duration of the recording. The zebrafish swimming pattern and locomotion parameters were determined *via* analysis using the Smart tracking software.

### Three-Axis Maze Test and Behavior Analysis

In the three-axis maze (Figure 1) fish are required to navigate a route based on x (forward/backward), y (lateral) and z (depth) axes and is designed as a measure of spatial memory. The maze is constructed from white 0.25″ acrylic held together with acrylic epoxy and sealed with aquarium sealant. The maze consists of a 20 × 20 × 60-cm tank divided into five 12 × 20 × 20-cm chambers with a 7 × 7-cm window cut into the corner or center of each insert as shown in Figure 1. A floating NutraFin Max feeding ring (PetCo, Inc.) was attached to the end of the maze in the goal chamber. The walls and inserts of the maze are constructed of white acrylic to minimize any external visual cues that could be used by the fish as markers. In addition, the maze was uniformly illuminated from above to minimize shadows and visual cues external to the maze. With the inserts in place, fish swim from one chamber to another through the windows in the inserts to reach a food reward in the goal chamber. The order of inserts in the maze was constant throughout the experiment, to allow the route to remain constant until the feeding ring as shown in Figure 1.

The detailed specifications regarding the maze were as per the given standard protocol (Nasir et al., 2012). Transfer latencies (TL) were recorded from day-1 to day-10 post-PTZ administration. An inflexion ratio (IR–day-1) = (TL0-TL1)/(TL1), (IR-day-2) = (TL0-TL2)/(TL2) rest as follows was calculated, where TL0 is the initial latency(s) at day-0 and TL1 and TL2 is the latency(s) at the Day-1 and day-2 trial respectively. The IR was calculated as compared to day-0 to measure the amount of memory increase each day with the progression of days/treatment. The behavior recordings during seizure activity and the three-maze test were analyzed to track the locomotor patterns. Tracking of the locomotor pattern was done by using the computer software SMART v3.4-Panlab Harvard Apparatus®.

#### Training

The fish were food-deprived for 1 day prior to the start of both the training and testing periods and are not fed outside of the maze throughout the duration of the experiment. Training consisted of two back-to-back trials on the day before testing. During training, the inserts were removed from the maze; the fish were netted from their home tank and placed at the end of the maze opposite the feeding ring. A small amount of Tetramin flakes was placed in the feeding ring, and fish were trained to swim the length of the maze to receive food. The fish were permitted to feed for more than 30 s up to maximum 1 min before being netted and returned to their home tank. Training allows the fish to acclimatize to the testing apparatus and to learn the location of the feeding ring at the opposite end of the tank. Food-deprived fish learn to associate the testing apparatus with food and actively search for the feeding ring when the inserts will be present. Training is necessary in order to avoid novel tank anxiety effect in fish during the actual testing period. Food deprivation is part of the experimental procedure. The reason behind food-deprivation is that fish learn to associate the testing apparatus with food and actively search for the feeding ring when the inserts are present. After repeated trials fish learn the task to reach the feeding ring and eat the food (Nasir et al., 2012).

#### Testing

Testing consists of one trial per day for 10 consecutive days. Vehicle control animals will be administered I.P with vehicle agent first and then administered with distilled water before placing it in the maze. For the PTZ treated group, each fish was administered with an 80 mg/kg dose I.P daily prior to the testing period. The fish were habituated for 10 min after PTZ administration to check the memory test in the three-axis maze. For the KA-treated group, each fish was administered with 3 mg/kg dose at the start of the experiment on day-1. For the EMB treated group, the fish was pre-treated with EMB (0.156/0.312/0.625 mg/kg) daily prior to the administration of PTZ. After specific treatment, the fish are placed in the start chamber and the response latency to reach the feeding ring in the last chamber is recorded. Fish will be allowed to feed for more than 30 s maximum up to 1 min before being returned to their home tanks. If fish failed to complete the maze within 10 min, they are fed and returned back to the home tank.

#### Gene Expression

Gene expression studies were carried out to determine the expression level of several inflammatory genes such as CCL2, HMGB1, TLR4, IFN- γ, TNF-α and IL-1. All the brain samples were collected in ice-cold 200 μl TRIzol® reagent (Invitrogen, Carlsbad, CA, USA) and immediately stored at −80°C until further usage. The study was divided into three steps such as isolation of mRNA, synthesis of cDNA strand and then real-time PCR to estimate the level of genes expressed.

##### Isolation of RNA and First Strand cDNA Synthesis

The mRNA was isolated by following the manufacturer’s protocol. In brief, brain tissue was properly homogenized in TRIzol® reagent, mixed with chloroform and centrifuged at 13,500 rpm (revolutions per minute) for 15 min at 4°C. The upper aqueous supernatant was transferred into new tubes and isopropanol was added, mixed and was incubated for 10 min at room temperature and later centrifuged for 10 min at 13,500 rpm at 4°C. The supernatant was discarded, and the pellets were subjected to rinsing with 75% ethanol. Then, the pellets were left for air drying between 5 and 8 min of air drying. Finally, nuclease-free water was added to each tube to dissolve the mRNA pellet. The concentration and purity of the isolated mRNA were measured by using a NanoDrop Spectrophotometer. The mRNA samples were converted into cDNA using Omniscript Reverse-transcription Kit (QIAGEN) according to the manufacturer’s protocol.

All the primer sets were provided by Qiagen (NL).

CCL2: Dr_ccl2_1_SG QuantiTect Primer Assay (Cat no: QT02205763) TLR4: Dr_tlr4ba_va. 1_SG QuantiTect Primer Assay (Cat no: QT02198539).

IFN-G: Dr_ifng1-2_1_SG QuantiTect Primer Assay (Cat no: QT02064328).

TNF-α: Dr_tnf_1_SG QuantiTect Primer Assay (Cat no.QT02097655).

IL-1: Dr_il1rapl1a_1_SG QuantiTect Primer Assay (Cat no.QT02131850).

eef1a1b: Dr_eef1a1b_2_SG QuantiTect Primer Assay (Cat no.QT02042684).

#### Estimation of Neurotransmitters by LC/MS-MS

Glutamate, GABA, and Ach are significant neurotransmitters to study epilepsy and cognition. These neurotransmitters were analyzed using the LC-MS/MS technique. All the standard neurotransmitters were prepared in methanol (0.1% formic acid) as a stock solution of 1 mg/ml and were kept at 4°C until use. Standards for calibration were prepared from the original stock solution. Serial dilution from 100–2000 ppb was used for calibration. The brain was homogenized in 200 μl of ice-cold methanol (0.1% formic acid). The homogenate was vortex-mixed for 1 min and then centrifuged at 18,000*g* for 10 min at 4°C. Finally, the supernatant was pipetted and placed into vials for LC-MS/MS analysis.

LC-MS/MS was run on an Agilent 1,290 Infinity UHPLC, coupled with Agilent 6,410 Triple, Quad LC/MS, ZORBAX Eclipse plus C18 RRHD 2.1 × 150 mm, 1.8-micron (P/N 959759–902) auto-sampler system (Agilent Technologies, Santa Clara, CA, USA). The samples were separated on a SMol-RRHD-Eclipse-C18-8 (15) UHPLC-160129-00011-Pos-DMRM used at 30°C. The mobile phase consisting of 0.1% formic acid in water (Solvent A) and acetonitrile with 0.1% formic acid (Solvent B) was used with a gradient elution: 0–3 min, 50% B; 3–6 min, 95% B; 06–07 min, 95% B at a flow rate of 0.1 ml/min. ESI-MS/MS Conditions were set as follows: ESI ion source, positive ion polarity, gas temperature 325°C, drying gas flow 9.0 L/min, nebulizer pressure 45 psi, Vcap 4,000 V. MS acquisition of GABA, Glu, ACh was performed in electrospray positive ionization multiple reaction monitoring (MRM) mode.

### Statistical Analysis

The data were analyzed by GraphPad Prism v.7.02 (San Diego, CA) with repeated measure (mixed model) two-way ANOVA followed by Bonferroni post-test to compare replicated means by row. Data are presented as means and standard errors of the mean (SEM) to assess the differences in seizure, latency and inflexion ratio. The results acquired for neurotransmitter levels and gene expression between treatments and control were analyzed by one-way ANOVA and subsequent Dunnett’s multiple comparison tests. A *p* of <0.05 indicates statistical significance.

## Results

### Daily Administration of PTZ Induces Chronic Epilepsy in Adult Zebrafish

**Figure 2:**
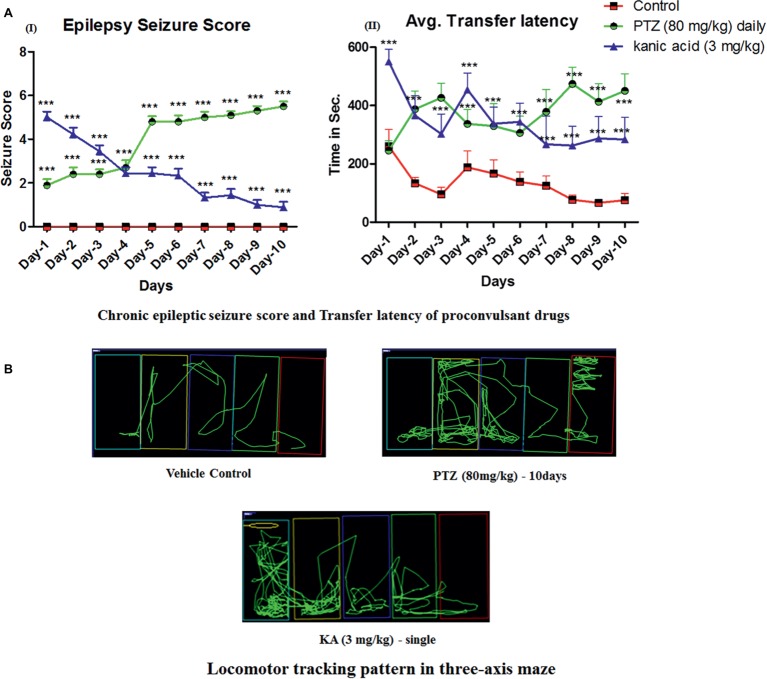
Daily administration of PTZ induces chronic epilepsy in adult zebrafish. **(AI)** Represents the seizure progression pattern of PTZ and KA from day-1 to day-10 with *F*-value of 413.7, DF-18 and mean square–19.41; **(AII)** represents transfer latency, the time taken by PTZ and KA-treated fish against a control group. PTZ (80 mg/kg) and KA–3 mg/kg significantly increase the time taken to reach the feeding ring. The data were analyzed by GraphPad Prism v.7.02 (San Diego, CA) with repeated measure (mixed model) Two-way ANOVA followed by Bonferroni post-test to compare replicated means by row. Data are presented as means and standard errors of the mean (SEM) to assess the differences in seizure, latency, and inflexion ratio: with *F*-value of 22.24, DF-18 and mean square–70,622 *p ≤ 0.05; **p ≤ 0.01; ***p ≤ 0.001. **(B)** Locomotor tracking pattern of a single representative fish in a treated group with the proconvulsant drug at day-10 in three-axis maze.

Daily administration of small doses of PTZ for 10 days induced full-blown seizures in zebrafish. It was observed that 80 mg/kg dose of PTZ cause a gradual increase in seizure scores from day-4 until day-10. Seizure score increase from score 1.5 to score 5 from day-1 to day-10. In addition, a single dose of KA-induced epileptic seizures, however, it was not able to maintain a high seizure score on daily observation and the score was eventually reduced from score 5 at day-1 to no seizure to score 1 at day-10 (Figure 2AI).

Moreover, in the three-axis maze, it was observed that all the fish from the control group showed a significant decrease in time taken to reach the feeding ring (goal chamber) from day-1 to day-10 and it was less than 100 s (Figure 2AII). However, all the fish from PTZ and KA-treated group found to worsen the cognitive function in zebrafish on observation of three-axis maze study. Indeed, the latency to reach the feeding chamber was high in PTZ and KA-treated fish when compared to the control group (Figure 2AII). It was observed that PTZ treated fish took more than 450 s to reach the feeding ring until day-10. However, the time was increased to 250 s in KA-treated fish when compared to the control group as shown in Figure 2AII.

### Daily Administration of EMB Ameliorates PTZ Seizures and Memory Decline

**Figure 3:**
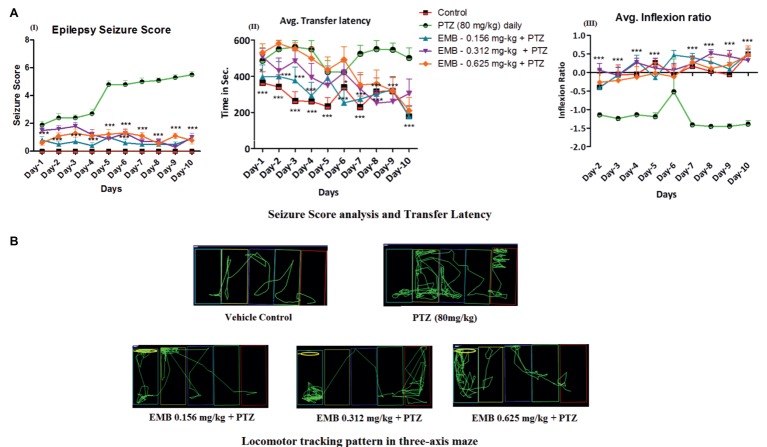
Daily administration of EMB ameliorates PTZ seizures and memory decline. **(AI)** Represents the seizure progression pattern of EMB against PTZ kindling from day-1 to day-10 with *F*-value of 106.4, DF-36 and mean square–5.489. **(AII)** Represents transfer latency, the time taken by EMB treated fish against PTZ treated fish. **(AIII)** Represents the inflexion ratio of EMB treated group compared to the PTZ treated group. The data were analyzed by GraphPad Prism v.7.02 (San Diego, CA) with repeated measure (mixed model) Two-way ANOVA followed by Bonferroni post-test to compare replicated means by row. Data are presented as means and standard errors of the mean (SEM) to assess the differences in seizure, latency and inflexion ratio with *F*value of 9.850, DF-36 and mean square–41,046, *p ≤ 0.05; **p ≤ 0.01; ***p ≤ 0.001. **(B)** Locomotor tracking pattern a single representative fish in a treated group with EMB against PTZ treated, control and PTZ negative control at day-10 in a three-axis maze.

PTZ at 80 mg/kg, when administrated daily for 10 days, produces kindling like behavior with a continuous unprovoked seizure. Fish, when treated with EMB, ameliorates the gradual increase in seizure generated due to PTZ kindling (Figure 3AI). It was found that EMB-0.156 mg/kg to EMB-0.625 reversed chronic epilepsy induced due to PTZ kindling and maintained a low seizure score from day-1 to day-10. All the animals from PTZ treated group demonstrated disruption in cognitive functions when analyzed on the three-axis maze model (Figure 3AII). Due to the alteration in cognitive functions induced due to PTZ kindling, fish get lost in the maze compartment of the three-axis maze. The tracking pattern of PTZ administered fish demonstrated complex behavior patterns, as well as not being able to analyze the path towards the feeding ring and hence spending more time in each compartment. As seen in Figure 2, the time is taken, and distance traveled by PTZ kindled fish to reach the feeding ring was higher as compared to the control group. Simple and straight tracking pattern was observed in the control group which might be due to the continuous daily trial of control fish in the three axis maze for 10 days. As the fish was treated daily with a single dose of EMB prior to PTZ administration, EMB ameliorated the epileptic seizure generated from PTZ kindling and improved the locomotor tracking pattern. EMB pre-treated fish with a dose ranging from 0.156 to 0.625 mg/kg demonstrated simple and improved tracking patterns similar to the control group on the 10th day of three-maze trial shown in Figure 3. Due to PTZ kindling effects, epileptic fish exhibited loss in memory function which is detrimental for egocentric navigation. EMB (0.156–0.625 mg/kg) significantly improved maze navigation times and reduced performance errors in the three-axis maze with P-value significant *p ≤ 0.05, **p ≤ 0.01, and ***p ≤ 0.001.

### Estimation of Neurotransmitters by LC/MS-MS

**Figure 4:**
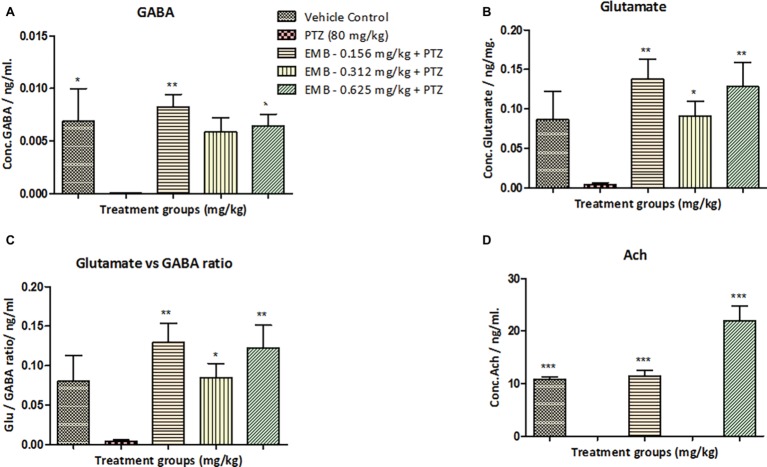
Neurotransmitters analysis in zebrafish brain after 10 days of three-axis maze trial. **(A)** Represents the concentration of GABA in the zebrafish brain. **(B)** Represents concentration of glutamate in the zebrafish brain with *F*-value = 3.976, R-square = 0.4429, *p* = 0.0156. **(C)** Represents the ratio of glutamate over GABA showing a significant increase in glutamate levels against PTZ treated group with *F*-value = 5.5.4, R-square = 0.5240, *p* = 0.0037. **(D)** Represents concentration of acetylcholine (Ach) in the zebrafish brain with *F*-value = 41.86, R-square = 0.9331, *p* = 0.0001. In each neurotransmitter analysis, all the control and EMB treated groups are compared with negative control PTZ treated group. Data are represented as mean ± SEM, *n* = 5 and statistically analyzed by one-way ANOVA followed by Dunnett’s test *p ≤ 0.05, **p ≤ 0.01, and ***p ≤ 0.001.

Neurotransmitter analysis was performed to check the amount of chemical change in the brain after chronic epilepsy treatment. Neurotransmitter analysis by LC/MS-MS demonstrated a significant reduction (^*^ *p <* 0.05) in the level of GABA in the PTZ administered group when compared to the control groups. Significant elevation in the level of GABA was observed on the EMB (0.156 mg/kg) (^**^ *p <* 0.01) and the EMB (0.625 mg/kg) (^*^ *p <* 0.05) administered groups when compared to the PTZ group (Figure 4). The level of glutamate was non-significantly different between the control group and the PTZ treated group. However, there was a significant elevation in EMB (0.156 mg/kg) (^**^ *p <* 0.01), EMB (0.312 mg/kg) (^*^ *p <* 0.05) and the EMB (0.625 mg/kg) (^**^ *p <* 0.01), administered group when compared to PTZ injected group. However, the brain Ach level was significantly decreased on the PTZ administered group as compared to the control group. In addition, there was significant upregulation in the level of Ach in EMB (0.156 mg/kg) (^***^ *p <* 0.001) and the EMB (0.625 mg/kg) (^***^ *p <* 0.001) treated group when compared to the PTZ treated group shown in Figure 4.

### EMB Pre-treatment, Reverse Epilepsy That Affects Expression Levels of Several Inflammatory Genes

**Figure 5:**
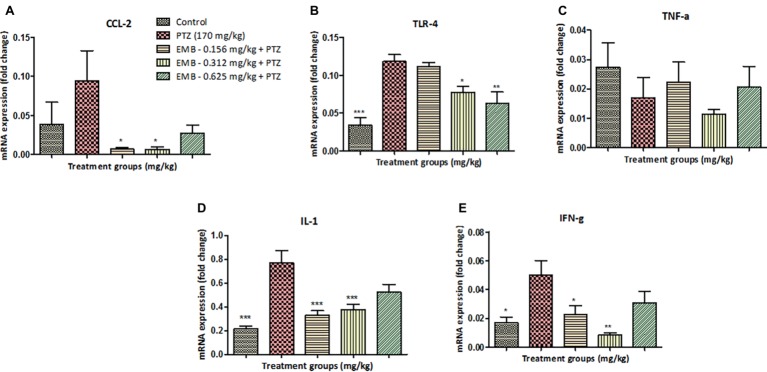
EMB pre-treatment, reverse epilepsy that affects expression levels of several inflammatory genes. **(A)** Represents graph plot for CCL2 mRNA expression in the zebrafish brain with *F*-value = 3.564, R-square = 0.4712, *p* = 0.0292. **(B)** Represents graph plot of TLR-4 mRNA expression in the zebrafish brain with *F*-value = 12.98, R-square = 0.7644, *p* = 0.0001. **(C)** Represents graph plot for INF-γ mRNA expression in the zebrafish brain with *F*-value = 5.414, R-square = 0.5751, *p* = 0.0059. **(D)** Represents graph plot of IL-1 mRNA expression levels in the zebrafish brain with *F*-value = 12.4, R-square = 0.7121, *p* = 0.0001. **(E)** Represents graph plot of TNF-α mRNA expression in the zebrafish brain with *F*-value = 1.127, R-square = 0.2198, *p* = 0.3788. In each gene, all the control and EMB treated groups are compared with negative control PTZ treated group. Data are represented as mean ± SEM, *n* = 5 and statistically analyzed by one-way ANOVA followed by Dunnett’s test *p ≤ 0.05, **p ≤ 0.01, and ***p ≤ 0.001.

CCL2 mRNA expression level was non-significantly upregulated in the PTZ treated group when compared with the control group. However, CCL2 mRNA expression level was significantly down-regulated in EMB (0.156 mg/kg) (^*^ *p <* 0.05) and the EMB (0.312 mg/kg) (^*^ *p <* 0.05) treated group as compared to the PTZ administered group. In addition, there was non-significant downregulation in the CCL2 mRNA expression level on EMB (0.625 mg/kg) treated group when compared to the PTZ treated the group as shown in Figure 5A.

TLR4 mRNA expression was significantly (^***^ *p <* 0.001) decreased in the control group when compared to the PTZ treated group. When compared to the PTZ administered group, TLR4 mRNA expression level was decreased in EMB (0.312 mg/kg) (^*^ *p <* 0.05) and the EMB (0.625 mg/kg) (^**^ *p <* 0.01). However, non-significant downregulation of TLR4 mRNA expression level was observed in the EMB (0.156 mg/kg) treated group when compared to the PTZ treated group as shown in Figure 5B.

TNF-α mRNA expression was non-significantly upregulated in the control group as compared to the PTZ treated group. However, no significant difference was observed in the TNF-α expression levels in all the EMB (0.156, 0.312 and 0.625 mg/kg) treated group when compared to PTZ treated group as shown in Figure 5C.

IL-1 mRNA expression was found to be significantly (^***^ *p <* 0.001) down-regulated in the control group when compared with the PTZ administered group. An IL-1 mRNA expression level was significantly (^***^ *p <* 0.001) down-regulated in the EMB (0.156 and 0.312 mg/kg) treated group as compared to the PTZ treated group. However, non-significant downregulation of the IL-1 mRNA expression level was observed in EMB (0.625 mg/kg) treated group when compared to the PTZ treated the group as shown in Figure 5D.

Significant (^*^ *p <* 0.05) downregulation in the level of INF-γ mRNA expression was observed in the control group as compared to the PTZ treated group. However, INF-γ mRNA expression was found to be significantly downregulated in EMB (0.156 mg/kg) (^*^ *p <* 0.05) and in the EMB (0.312 mg/kg) (^**^ *p <* 0.01) the treated group as compared to the PTZ treated group. In comparison with the PTZ administered group, non-significant downregulation in INF-γ mRNA expression levels was observed in the EMB (0.635 mg/kg) treated group as shown in Figure 5E.

## Discussion

The development of new drug treatment for chronic epilepsy induced cognitive dysfunction has largely stalled with very minor advances over the past few decades (Witt and Helmstaedter, 2017). In fact, in addition to minimizing seizures, the currently available AEDs produces cognitive alterations in around one-third of the epileptic patients (Witt and Helmstaedter, 2017). Herein, current study utilizes multifaceted approach and for the very first time develop PTZ kindling induced chronic epilepsy which significantly altered memory as compared to KA. We also evaluated the therapeutic potential of EMB against PTZ kindled chronic epilepsy and related cognitive alterations. In addition to that, modulation of several neurotransmitters and inflammatory genes by EMB treatment of varying doses (0.156, 0.312 and 0.612 mg/kg) has opened new therapeutic approach to deal with epilepsy and related memory problems (van Vliet et al., 2018).

Chronic epilepsy is a complex brain disorder exhibiting multiple underlying known and unknown causes with poorly understood mechanisms (Staley, 2015). Impairment of learning and memory is frequently observed in long-term epileptic patients and chronic epilepsy (Paudel et al., 2018b). To the best of our knowledge, current options to treat epilepsy only help with seizures and do not help to improve cognitive impairment, or standard AEDs contribute to impaired memory in patients with epilepsy (Santulli et al., 2016). In relation to that, current investigation shed light on the development of natural product-based novel therapy of using pro-inflammatory targets that can ameliorate chronic seizure as well as associated cognitive alterations (Barker-Haliski et al., 2017).

In spite of growing interest of utilizing zebrafish as an experimental model, much more remained to learned about the spatial cognitive abilities of zebrafish in the epileptic condition as compared to more widely used mammalian species (Meshalkina et al., 2017a). Zebrafish is one of the most emerging model systems to study neurologically related disorders and memory function (Fontana et al., 2018). Use of zebrafish as a model system is considered to be one of the most highly productive animal models with low cost, ease in doing an in-vivo pharmacodynamic study and easy tracking analysis of behavior study (Khan et al., 2017). Recently, a large number of studies related to PTZ-kindling are conducted on rodents which have high cost and is a time-consuming procedure (Kumar et al., 2016; Shimada and Yamagata, 2018). It is also known that once the rodents and large animals are epileptic they become difficult to handle, which is exactly opposite to zebrafish model (Meshalkina et al., 2017b). Zebrafish are easy to handle and inject, they are cost-effective, easy to maintain and feed, can adapt to new conditions quickly and produce a robust accurate result (Kearney, 2018). Moreover, zebrafish as an experimental model has been significantly important in the investigation elucidating epilepsy-related cognitive alterations (Stewart and Kalueff, 2012; Kundap et al., 2017).

Seizure-like behavioral and neurophysiological responses can be evoked in adult zebrafish by various genetic modifications and pro-convulsive chemicals that collectively strengthen the growing utility of this model for studying epilepsy (Kalueff et al., 2014). Moreover, learning and memory function can be tested in the zebrafish using various types of mazes like the T or Y axis maze, light/color preference test and three-axis maze (Nasir et al., 2012). In the neurological translational research, many advances have been made in understanding CNS and epilepsy-related problems, but there is still a lack of an animal model that can fully recapitulate the clinical phenotypes of human epilepsy-related cognitive dysfunction. In this regard, there is an increased understanding about the usability of zebrafish as an animal model in epilepsy research and has been demonstrated the features of human epilepsy (Mussulini et al., 2013, 2018).

PTZ (80 mg/kg) kindling acts *via* GABA_A_ receptor induces epileptic seizures and disrupts the cognitive function for 10 days in adult zebrafish as observed on the three-axis maze test. Repeated administration of PTZ produces a chronic epilepsy-like condition in zebrafish along with the significant loss of cognitive performance in one of the three-axis mazes which is considered to be one of a complex maze. Repeated administration of small doses of PTZ (80 mg/kg) successfully induces a chronic epilepsy-like condition at least for 10 days as evidenced by increased seizure score. These findings were in agreement with an earlier reported study conducted using PTZ in a rat model reporting impaired memory and epileptic seizures (Golechha et al., 2010). Similar findings were reported earlier regarding PTZ kindling in rodents, where administration of PTZ resulted in a progressive increase in sensitivity of epileptic seizures (Löscher, 2017). Similarly, in the present study, a single dose of KA (3 mg/kg) failed to maintain the progressive increase in seizure sensitivity throughout the experiment. This finding was different from the findings from rodents where a single high dose of KA produces full-blown seizure (Demars et al., 2018). This might be due to the virtue (ability of excellence or performance) of zebrafish and its regenerative property which regenerates the damaged neurons and makes the fish less sensitive (Ceci et al., 2018). These findings implicate that a single dose of KA (3 mg/kg) lack (reduced in sensitivity or less sensitive) produce long term sensitivity in zebrafish for epileptic seizures.

The cognitive behavior of PTZ treated fish showed that latency to reach the feeding ring was high in PTZ treated fish as compared to the control group. It might be because PTZ treated fish could not find their way to the feeding ring and get repeatedly lost and backtrack to the previous compartment instead of moving forward to the feeding ring. This implies that PTZ kindling impairs memory in adult zebrafish. Interestingly, latency to reach the feeding ring in KA-treated fish was less than the PTZ treated group but was higher than the control group. This implies that though a single dose of KA (3 mg/kg) does not maintain a chronic epilepsy-like stage for 10 days, it still manages to disrupt the memory function for 10 days. All these findings are in agreement with the earlier findings reporting cognitive alteration in epileptic conditions (Black et al., 2010; Leeman-Markowski and Schachter, 2016). We observed that pre-treatment with different EMB doses rescued the fish from the epileptic stage as well as ameliorating its cognitive function. In addition, pre-treatment also reduces seizure scores and seizure intensity until day 10 as compared to PTZ treated fish. This finding strengthens the usability of EMB against chronic seizure-induced cognitive disruptions. However, evaluating the ameliorative potential of EMB against a range of seizure models and related cognitive impairments would further verify this statement.

Neurotransmitters play a crucial role in regulating neuronal excitation and maintaining normal behavior of the cognitive function (Moavero et al., 2017). Hypoactivity GABA leads to the increase in higher dopamine release, causing dopaminergic neurons to influence the GABAergic system to shut down through the GABA_A_ receptor (Werner and Coveñas, 2011). Moreover, glutamate hyperactivity is influenced by n-methyl-D-aspartate (NMDA) receptor, inhibiting serotonin release *via* postsynaptic glutaminergic receptors, which can induce epileptic seizures (Lewerenz and Maher, 2015). In the current investigation, in the EMB treated group the level of GABA was found to be significantly rescued back to control level as compared to the PTZ treated group, implicating the release of dopamine causing epileptic seizures. Also, an increase in the level of GABA in EMB treated group helps in controlling epileptic seizures. On the other hand, the level of glutamate was found to be elevated in the EMB treated group in comparison to the PTZ treated group, however, it does not affect epileptic behavior because the inhibitory GABA was controlling the hyperexcitation of neurons from glutamate (Medrihan et al., 2014). Ach has a crucial role in regulating attention, learning and short-term memory (Klinkenberg et al., 2011). Moreover, Ach has been implicated in controlling the release of glutamate in the brain during epileptic activity (Belousov et al., 2001). The current study documented the increased level of Ach in the EMB treated group compared to the PTZ treated group and reported a similar line of neurotransmitter levels as observed in our earlier studies after PTZ administration (Kundap et al., 2017).

The alteration of neurotransmitter is closely associated during the epileptic event. Many studies have shown the drastic increase in the level of inhibitory neurotransmitters like GABA in the epileptic brain (Swaminathan et al., 2018). The study suggests that proinflammatory biomarkers contribute to regulation of epilepsy-associated biochemical changes CNS. The neuroinflammatory markers like IL-1 TNF-α and INF-ϒ play an important role in regulating GABA expression and transportation of extracellular GABA in the brain (Su et al., 2015). The TLR-4 and RAGE inflammatory pathway is stimulated by one of the most important pro-inflammatory cytokines knows as HMGB1, which is responsible for the release of glutamate and causing hyperexcitability of the brain during epilepsy (Paudel et al., 2019).

There is an increased understanding of the role of inflammation in epileptic seizure (Vezzani and Granata, 2005; Vezzani et al., 2013). Inflammatory mediators are produced by glia, neurons, endothelial cells of the blood-brain barrier (BBB), and peripheral immune cells and might contribute to the onset and perpetuation of seizures in various types of epilepsy (Liu and Huang, 2011). Induction of recurrent seizures or single prolonged seizures by chemoconvulsants or electrical stimulation triggers rapid induction of inflammatory mediators in brain regions and upregulates CCL2 expression (Vezzani et al., 2011). Moreover, findings are emerging implicating the same inflammatory pathways in epilepsy and related neurobehavioral comorbidities including cognitive dysfunctions (Mazarati et al., 2017; Paudel et al., 2018b). Current study reported an increased level of CCL2 in PTZ kindled group. These findings are in corroboration with earlier findings reporting upregulation of CCL2 in the epileptic brain (Bozzi and Caleo, 2016). Interestingly, EMB treatment reduces the expression level of CCL2 as compared to the PTZ treated group. These findings are in agreement with the earlier findings reporting anti-inflammatory activities of EMB (Lee et al., 2018). These results are in line with the recent finding of anti-inflammatory activity of EMB in A549 cells and epithelial cell line (Ahmed, 2017).

TLR4 is the principal receptor for HMGB1 and has been implicated in seizure generation (Iori et al., 2017). The contribution of TLR4 in seizure generation is more than that of RAGE (Iori et al., 2013). This makes TLR4 worth exploring against several seizure models in order to elucidate the contribution of TLR4 in seizure generation. Similar to earlier findings (Maroso et al., 2010), we observed an increased level of TLR4 in the PTZ administered group suggesting its role in a seizure. EMB pre-treatment reduces the expression level of TLR4 which might be due to the prevention of epileptic seizure by EMB pre-treatment. The increased level of HMGB1 and TLR4 after pro-convulsant administration in current investigation supports the notion that HMGB1-TLR4 signaling may contribute to generating and perpetuating seizures (Maroso et al., 2010).

The effect of TNF-α on seizures depends mainly on its endogenous brain levels and the receptor subtypes predominantly stimulated by this cytokine (Vezzani and Granata, 2005). Few studies have reported the seizure inhibiting activity of TNF-α mainly *via* p75 receptors. Generally, the levels of TNF-α found to be elevated in seizure conditions, either in rodents (Ashhab et al., 2013) or in adult zebrafish (Choo et al., 2018). Surprisingly, we currently observed a non-significant decrease in the level of TNF-α after PTZ administration. However, this finding is in corroboration with the earlier reported study, reporting a decrease in TNF-α levels even after the pro-convulsant (pilocarpine) administration in rats (Marchi et al., 2007).

It was reported that IL-1 mediates upregulation of NMDA receptor in PTZ induced seizures causing epileptogenesis in the rat model (Yi et al., 2017). In an experimental model of status epilepticus (SE) triggered by electrical stimulation, brain mRNA expression level of IL-1 has been elevated (De Simoni et al., 2000). In a similar line, brain mRNA expression level of IL-1 was upregulated after PTZ administration. On the other side, EMB pre-treatment reduced the brain mRNA expression level of IL-1 suggesting its plausible anti-inflammatory potential.

IFN-γ plays an important role in the development of the brain’s excitatory seizure pathways, and it has been associated with the development of limbic seizures (Borham et al., 2016). IFN-γ has been implicated in the seizure generation; this makes IFN-γ worth exploring in the current investigation. We observed an increased level of IFN-γ after PTZ administration, which is in support of an earlier study reporting elevated IFN-γ level in epileptic condition (Mao et al., 2013). Interestingly, EMB pre-treatment decreases the level of IFN-γ which might be since EMB pre-treatment prevents the epileptic seizure which in turn helps in preventing neuroinflammation. These overall findings from the current study suggest the possible anti-inflammatory potential of EMB in addition to seizure retarding and related memory improving potential. More studies describing the fact that embelin in the absence of PTZ or seizures can reduce neuroinflammation can be the point of interest for further researchers.

## Conclusion

Herein, we conclude that EMB suppresses seizure-like behavior and improves cognitive function altered due to a chronic epilepsy condition. EMB significantly modulates inflammatory genes affected by seizure and also affects neurotransmitter levels, which eventually improves the cognitive status of the animals. Moreover, behavioral observation, neurotransmitter analysis and gene expression results suggest that EMB can reverse learning and memory dysfunction associated with chronic epilepsy in a zebrafish model. Though we developed a KA-induced seizure model, we did not assess the ameliorative effect of embelin against KA-induced seizure and related cognitive alterations as a single KA dose did not produce chronic epilepsy like behavior similar to PTZ kindling. We acknowledge this part as a limitation of the current study. However, overall findings suggest that EMB has potential therapeutic value for the management of epilepsy and related cognitive alterations in experimental studies. However, further work is highly recommended to evaluate the therapeutic effects of EMB against genetic models and to compare its efficacy with current AEDs.

## Ethics Statement

The experimental protocol was approved by the MARP Animal Ethics Committee, Monash University, Australia (MUM/2017/03 and MARP/2017/003).

## Author Contributions

UK performed the experiment, analysis of the results and writing of the manuscript. YP contributed to manuscript writing, result analysis and proofreading. YK contributed to the gene expression study and result analysis. IO contributed to LC-MS/MS study and result analysis. MS conceptualized the idea, contributed in the designing of the study, result interpretation & analysis, editing and proofreading of the manuscript. All authors have approved the final version of the manuscript.

### Conflict of Interest Statement

The authors declare that the research was conducted in the absence of any commercial or financial relationships that could be construed as a potential conflict of interest.
